# Concurrent myasthenia gravis-related cervical thymoma in a patient with diffuse large B-cell lymphoma: a case report 

**DOI:** 10.1186/s13256-021-03225-2

**Published:** 2022-01-12

**Authors:** Chutima Kunacheewa, Sattawut Wongwiangjunt, Sanya Sukpanichnant

**Affiliations:** 1 Department of Medicine, Faculty of Medicine Siriraj Hospital, Mahidol University, Bangkok, Thailand; 2 Department of Pathology, Faculty of Medicine Siriraj Hospital, Mahidol University, Bangkok, Thailand

**Keywords:** Cervical thymoma, Diffuse large B-cell lymphoma, Misdiagnosis, Myasthenia gravis

## Abstract

**Background:**

Cervical thymoma is a rare thymic epithelial neoplasm. Evidence supports an increased risk of second primary malignancies in patients with thymoma. We report a rare case of a patient with synchronous cervical thymoma and diffuse large B-cell lymphoma.

**Case presentation:**

An 81-year-old Thai woman was referred for further treatment of diffuse large B-cell lymphoma at Siriraj Hospital, Bangkok, Thailand. While waiting for a review of the original pathological examination of a mass in the left neck and a mass in the left arm, the attending physician noticed ptosis of the upper eyelids, which was proven to be caused by myasthenia gravis. The final pathology review confirmed that the arm mass was diffuse large B-cell lymphoma, but the neck mass was cervical thymoma, type B1, not diffuse large B-cell lymphoma. Interestingly, the patient reported that the arm mass had been present for 2 years, while the neck mass had grown rapidly in the past month. A diagnostic challenge had arisen when the initial morphological evaluation was not performed with care, causing the first pathologist to misinterpret that the neoplastic cells in both masses were the same.

**Conclusion:**

Concurrent cervical thymoma and diffuse large B-cell lymphoma were proven after a careful pathology review, leading to better clinical management.

## Background

Cervical thymoma is a rare neoplasm of the thymic epithelium, which arises in an ectopic thymus that originated from an undescended thymus during embryonic development. An increased risk of developing second primary malignancies has been observed synchronously and metachronously in patients with thymoma [1, 2]. It is, therefore, possible to have concurrent cervical thymoma and malignant lymphoma. From a clinical point of view, myasthenia gravis (MG) should be an important clue to an underlying thymoma in such a case. This is because MG is found in 50% of classic cases of thymoma and 10% of patients with ectopic thymoma [2]. However, it will be a diagnostic challenge for a pathologist who receives two masses for pathological examination—one excised from the lateral side of the neck and the other from the forearm—without knowing that the patient had MG. If the morphology shows diffuse infiltration by noncohesive mononuclear cells, there will be a high chance of the pathologist diagnosing the two masses as malignant lymphomas. The clinical management should be different if the patient is diagnosed with concurrent cervical thymoma and malignant lymphoma.

In this paper, we report a case of MG related to cervical thymoma and concurrent diffuse large B-cell lymphoma (DLBCL) in an 81-year-old woman. She was initially diagnosed with DLBCL involving two masses, one on the left side of the neck and the other on the left forearm. However, the patient was found to have MG-related cervical thymoma after pathologic review, while the forearm mass was confirmed to be DLBCL. Pathologic and hematologic points of view are discussed. The rarity of cervical thymoma and its histological similarities with type B1 thymoma (a predominance of lymphoid cells) present diagnostic challenges for pathologists and hematologists.

## Case presentation

An 81-year-old Thai woman presented to her local hospital with generalized lymphadenopathy for 3 months, a 5 × 3 cm left forearm mass (stable for 2 years), and a 3 cm left neck mass that developed rapidly over 3 months concurrently with appetite loss. Laboratory investigations showed a normal complete blood count and blood chemistry. After surgical excision, both masses were pathologically diagnosed as DLBCL; the woman was referred to Siriraj Hospital for definite treatment.

During the month preceding her Siriraj appointment, another left forearm mass grew rapidly near the previously excised area. Physical examination at Siriraj revealed a 7 × 5 cm left epitrochlear lymph node (LN) and a 2 cm right inguinal LN. Noticing ptosis of the left eyelid, the attending physician suspected a paraneoplastic syndrome such as MG. The patient reported that she had diplopia and progressive dysphagia before the excisions, with concurrent development of hoarseness during lengthy conversations. Her left palpebral fissure (0.3 mm) was narrower than the right (1 cm) and she had limited left upward gaze. Antiacetylcholine receptor antibody testing was positive, and electromyography revealed postsynaptic neuromuscular junction disorder. A neurologist confirmed oculobulbar MG. A chest X-ray failed to show any mediastinal mass for thymoma.

### Pathological materials for review

The original slides and corresponding tissue blocks were received for review along with a photocopy of the pathology report for verification. The gross examination record showed a mass in the left neck (9 × 5 × 4 cm) and a mass in the left arm (8 × 3 × 2.8 cm). According to the original pathology report, both masses had a diffuse, grayish-white, cut surface, and they were composed of abnormal large lymphoid cells with oval-to-round vesicular nuclei, conspicuous nucleoli, and scant cytoplasm, together with some admixed small- and medium-sized lymphoid cells in the background. Immunostained slides of the left arm mass were received for review, including CD3, CD20, cyclin D1, BCL2, and MUM1.

### Pathology

**Fig. 1:**
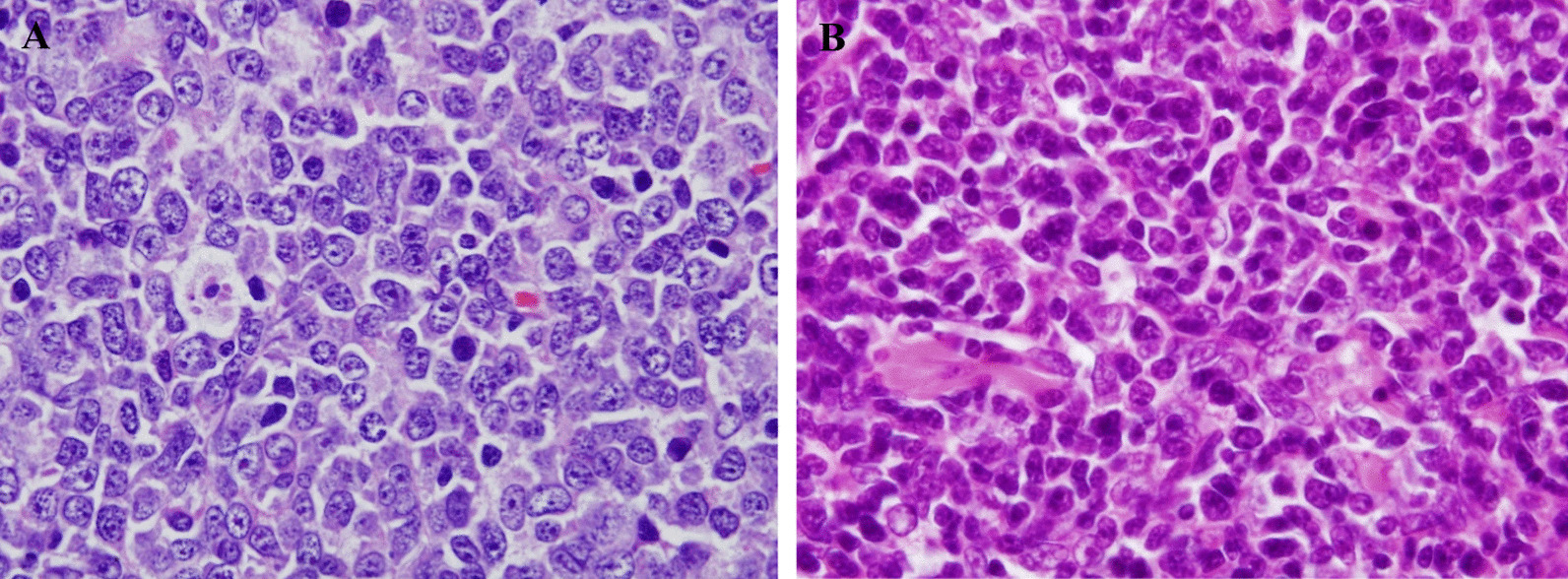
Comparison of the large lymphoma cells in the left forearm mass **A** and the cortical thymocytes in the left neck mass (**B**). Note the large size and distinct nucleoli of the large lymphoma cells, whereas the smaller cortical thymocytes in the thymoma have inconspicuous nucleoli and more delicate nuclear chromatin

**Fig. 2:**
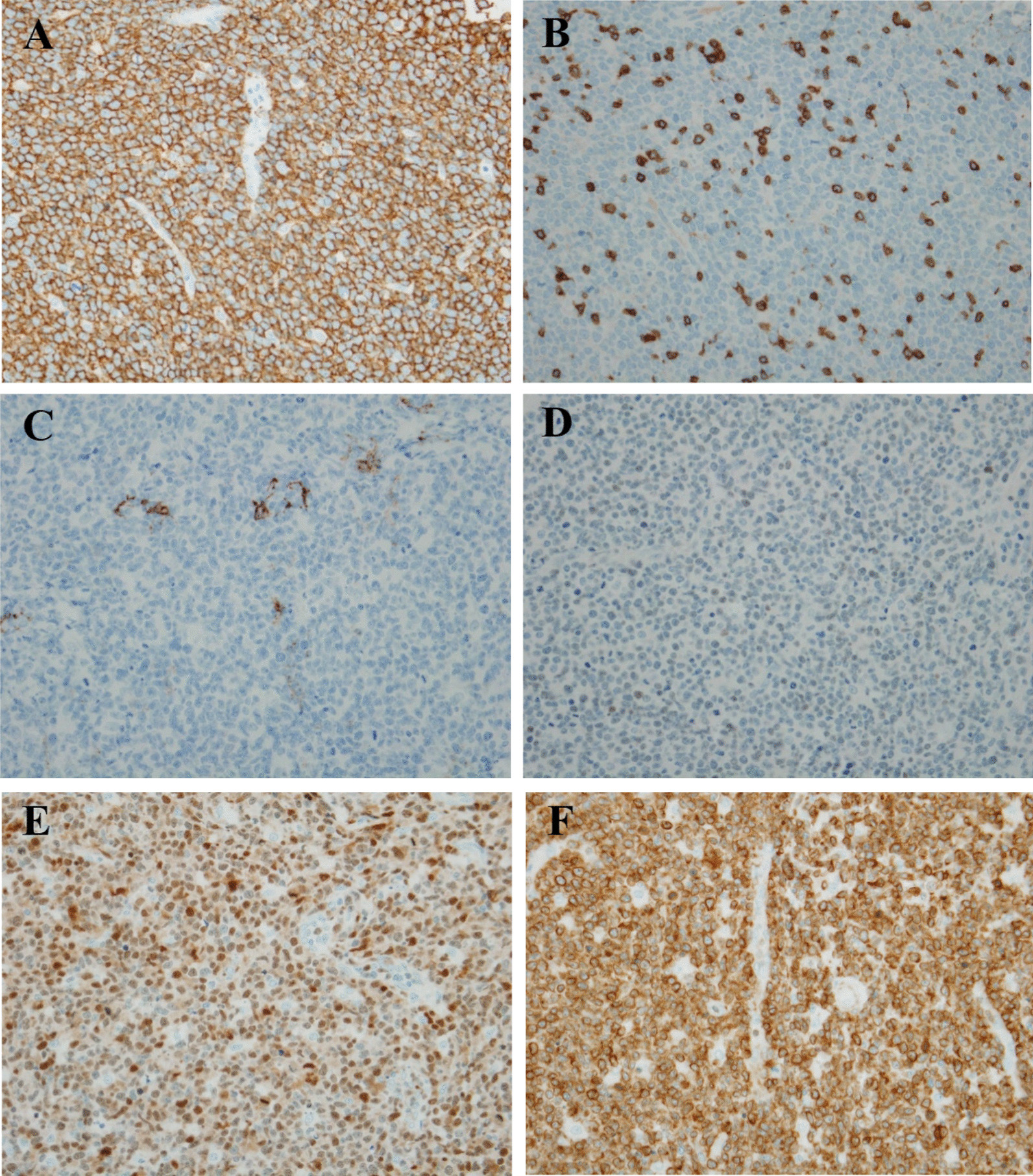
Compilation of the pathological findings of diffuse large B-cell lymphoma in the left forearm mass. **A** CD20 (+); **B** CD3 (−); **C** CD10 (−); **D** BCL6 (−); **E** MUM1 (+); and **F** BCL2 (+)

**Fig. 3:**
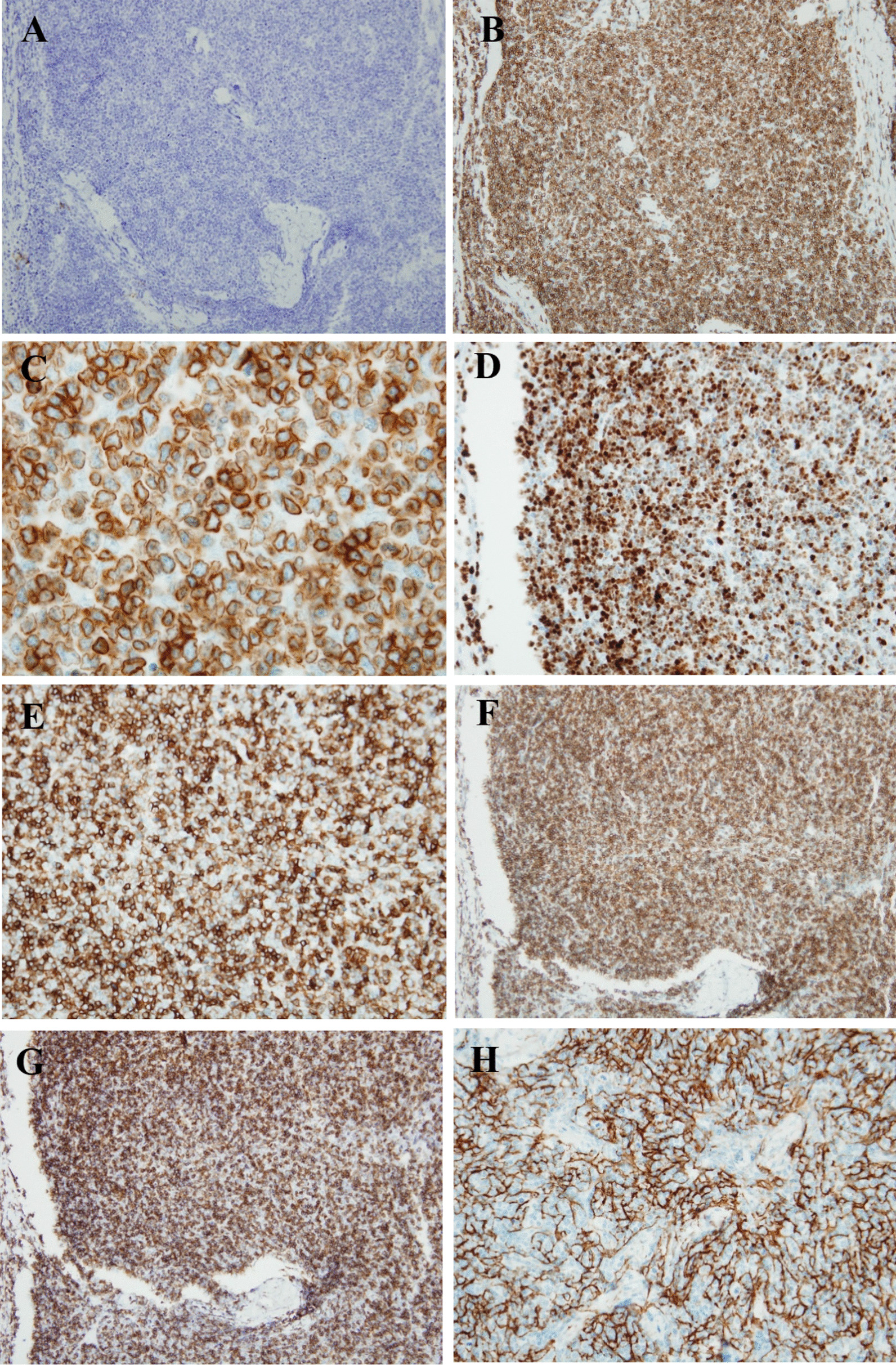
Compilation of the pathological findings of type B1 thymoma in the left neck mass. **A** CD20 (−); **B** CD3 (+); **C** cytoplasmic CD3 (+); **D** TdT (+); **E** CD1a (+); **F** CD4 (+); **G** CD8 (+); and **H** AE1/AE3 (+), reticular pattern of neoplastic thymic epithelial cells

Pathologic review was performed. The mass of the left forearm was confirmed to be a typical DLBCL (Fig. 1A). Lymphoma cells were large B-cells of the nongerminal center B-cell phenotype (CD20+ CD10− BCL6− MUM1+; Fig. 1A). They expressed BCL2 protein and had a high Ki-67 proliferation index (90%; Fig. 2). The neck mass comprised smaller cells with more delicate nuclear chromatin and small nucleoli. They were intermingled with elongated cells with delicate nuclear membranes, more open nuclear details, and inconspicuous cytoplasmic borders (Fig. 1B). Initially, without knowing the growth rate of each mass or the presence of MG in the patient, the neck mass was suspected to be an indolent B-cell lymphoma; however, immunostaining did not reveal lymphoma B-cells. The small cells were predominantly CD3+ with a cytoplasmic pattern and a high Ki-67 proliferation index (90%). They expressed TdT, CD1a, CD4, and CD8, indicating an immature T-cell phenotype and suggesting T-cell lymphoblastic lymphoma (T-LBL). Nevertheless, immunostaining for AE1/AE3 cytokeratins showed the typical reticular pattern of neoplastic thymic epithelium, confirming that the neck mass contained type B1 thymoma (Fig. 3). To distinguish metastatic thymoma from cervical LN and cervical thymoma, immunostaining for D2-40 was performed. It confirmed that the neck mass was the cervical thymus as there were no D2-40+ lining cells for the LN subcapsular sinuses. Hence, the patient had cervical thymoma and a concomitant forearm tumor with DLBCL. A review of the imaging findings confirmed that she did not have any mediastinal mass.

### Clinical course

There was no marrow involvement. Computed tomography found a right interlobar LN (1.4 cm in diameter). The patient did not receive standard treatment for DLBCL [an anti-CD20 monoclonal antibody (rituximab) plus CHOP (R-CHOP) regimen] as she was not eligible to access R-CHOP. Instead, she received mini-CHOP (cyclophosphamide, doxorubicin, vincristine, and dexamethasone), which produced a very good response. She achieved complete remission after the sixth chemotherapy course. The cervical thymoma was in a limited stage and was totally excised at the first operation. Her oncologist recommended 6-monthly computed tomography scans. The MG was controlled with pyridostigmine and prednisolone. Currently, 14 months after the diagnosis and 8 months after the CHOP regimen, she is still in remission for both thymoma and lymphoma.

## Discussion

From a pathological viewpoint, diagnosing this patient with concurrent cervical thymoma and DLBCL was challenging, and it highlights the need for careful histological evaluation and high-quality histological sections stained with H&E. The initial local hospital misdiagnosis of both masses having DLBCLs resulted from immunostaining having only been conducted on the forearm mass. Conversely, if immunostaining had only been performed on the neck mass, the cortical thymocytes in that mass might have been misinterpreted as T lymphoblasts, leading to a misdiagnosis of T-LBL.

If a pathologist suspects type B1 thymoma, immunostaining for AE1/AE3 cytokeratins will reveal the typical reticular pattern of neoplastic thymic epithelial cells needed for a definitive diagnosis. There is another potential pitfall for diagnosing a neck mass as metastatic thymoma or cervical type B1 thymus: pathologist–clinician communication is essential to ascertain whether a patient has a mediastinal mass. To discriminate between an LN and thymus, careful gross examination of tissue samples (including the capsule) is crucial. A lack of subcapsular sinuses will confirm the absence of an LN and the presence of an undescended cervical thymus. Immunostaining for D2-40, a marker for lymphatic lining cells, will determine whether a mass is an LN. As D2-40+ lymphatic lining cells were absent in our patient, the diagnosis was cervical type B1 thymoma.

From a hematologic viewpoint, concurrent cervical thymoma and lymphoma are rare. Thymoma patients have increased risks of autoimmune diseases and malignancies caused by T-cell dysregulation [1, 3], associated with T-cell stimulation by neoplastic thymus epithelial cells. In contrast, B-cell lymphoma is coincident with thymoma due to T-cell dysregulation, leading to uncontrolled B-cell growth.[1, 4] Only eight cases of concurrent thymoma and hematological malignancy have been published since 1990 (Table 1); all had anterior mediastinal masses. Our patient is the only reported case of cervical thymoma with MG concurrent with DLBCL.

**Table 1:** Concurrent thymoma and hematological malignancy cases reported between 1990 and 2020, including the present case

Author/year	Age/sex	Thymoma type	Hematological malignancy	Co-immune disorder	Clinical course
Szolkowska *et al*. 2017 [5]	52, male	B1/B2	SLL	–	CR at 2 years
Chen *et al*. 2015 [6]	74, male	A	DLBCL	–	CR at 7 months
Ito 2015 [7]	62, male	AB	T-LBL	–	CR at 1 year
Fraser *et al*. 2012	59, male	B1	PTCL, NOS	–	Death at 7 months
Yamato *et al*. 2006 [8]	59, male	B3	DLBCL	–	CR at 5 months
Rovera *et al*. 2003 [9]	65, male	AB	T-LBL	–	Death at 8 months
Khoury *et al*. 2003 [10]	62, male	NA	CLL/SLL	NA	NA
Friedman *et al*. 1994 [11]	95, male	NA	T-LBL	–	Death at 10 days
The present case, 2020	81, female	B1	DLBCL	MG	CR at 14 months

*CLL* chronic lymphocytic leukemia, *CR* complete remission, *DLBCL* diffuse large B-cell lymphoma; *MG* myasthenia gravis, *NA* not available, *PTCL* peripheral T-cell lymphoma, *NOS* not otherwise specified, *SLL* small lymphocytic lymphoma, *T-LBL* T lymphoblastic lymphoma/leukemia

Surprisingly, the forearm mass persisted for 2 years before growing rapidly. Possibly, it was an indolent lymphoma that completely transformed to DLBCL. It is not known whether the indolent lymphoma was not controlled by T-cell dysregulation when the cervical thymoma developed, leading to a complete transformation to DLBCL. Unfortunately, the forearm mass was not initially thoroughly sampled microscopically, and no remaining formalin-fixed tissue was available to detect indolent lymphoma.

To suspect and diagnose rare cases such as this correctly and early, one needs an experienced pathologist who can recognize the different morphologies of the mononuclear cells in the two masses. Additionally, type B1 thymoma should always be considered in the differential diagnosis of T lymphoblastic lymphoma. To this end, using immunostaining for AE1/AE3 cytokeratins will highlight the reticular pattern of neoplastic thymic epithelial cells. MG is an uncommon paraneoplastic syndrome in lymphoma; therefore, concurrent thymoma should be considered. The lack of a mediastinal mass in chest imaging should raise the possibility of ectopic thymoma (cervical thymoma in our case). Moreover, in patients who present with multiple masses that have a discordant response to standard chemotherapy, biopsy of the enlarged mass is recommended to confirm the diagnosis.

The treatment modality for patients with concurrent thymoma and malignant lymphoma depends on the staging of thymoma and the aggressiveness of concurrent lymphoma. If thymoma occurs locally, only surgery with or without local radiation is adequate to control the disease [5–9]. The treatment of lymphoma is inhomogeneous according to the type. Aggressive lymphomas such as DLBCL and T-LBL require systemic chemotherapy [6, 9]. Moreover, the level of fitness of a patient is an important consideration for decision-making on the chemotherapy regimen. The majority of reported cases were diagnosed at advanced age. Thymomas of these patients were excised; in some cases, this was followed by local radiation. Two patients with concurrent thymoma and DLBCL achieved complete remission after being treated with rituximab-based regimens plus multiple chemotherapy agents [6, 8].

## Conclusion

In any lymphoma patient with a mass showing different growth rate or discordant response to treatment from the other masses, reevaluation of the disease including careful pathological review is highly recommended as it is possible that the particular mass may be other disease, such as in the case report herein with concurrent thymoma and lymphoma.

## Data Availability

Not applicable.

## Abbreviations

DLBCL: Diffuse large B-cell lymphoma
LN: Lymph node
MG: Myasthenia gravis
T-LBL: T-cell lymphoblastic lymphoma
